# Prospective evaluation of vascular changes in acute respiratory infections in children with cystic fibrosis1The preliminary results of this study were presented as an oral presentation at the 3nd Middle East Cystic Fibrosis Conference(21-23 March 2019, Istanbul, Turkey).

**DOI:** 10.3906/sag-2002-61

**Published:** 2020-06-23

**Authors:** Gökçen KARTAL ÖZTÜRK, Aykut EŞKİ, Figen ÇELEBİ ÇELİK, Seçil CONKAR, Figen GÜLEN, Esen DEMİR, Ahmet KESKİNOĞLU

**Affiliations:** 1 Department of Pediatrics, Division of Pulmonology, Faculty of Medicine, Ege University, İzmir Turkey; 2 Department of Pediatrics, Division of Pediatric Nephrology, Faculty of Medicine, Ege University, İzmir Turkey

**Keywords:** Cystic fibrosis, arterial stiffness, pulse wave velocity analysis, augmentation index, pulmonary exacerbation

## Abstract

**Background/aim:**

Acute exacerbations and chronic inflammation are risk factors for cardiovascular disease (CVD) in cystic fibrosis (CF) patients. The aim of this study was to investigate the effects of acute exacerbation therapy on arterial stiffness in children with CF.

**Materials and methods:**

Augmentation index (Aix) and pulse wave velocity (PWV) were measured before and after treatment and 1 month after the end of treatment in patients with acute exacerbation. The relationship between hemodynamic measurements and c-reactive protein (CRP) and pulmonary function tests (PFTs) was investigated.

**Results:**

Measurements before and after treatment were evaluated in 27 patients and were repeated in 21 patients who were clinically stable 1 month following acute exacerbation. There was a significant decrease in CRP and an increase in spirometry parameters after treatment. While no significant difference was found between PWV (P = 0.33), a significant difference for Aix before (41.95 ± 12.96%) and after (30.95 ± 11.47%) treatment and before treatment and stable clinical condition (34.19 ± 14.36%) was obtained (P =0.00, and P =0.01, respectively). No significant difference in heart rate and other hemodynamic measurements was found. Pretreatment Aix is associated with poor clinical condition (PFTs, BMI, and clinical score) and systemic inflammation (CRP) (P <0.05).

**Conclusion:**

The decrease of arterial stiffness (Aix) with acute exacerbation treatment in children with CF has been demonstrated. This result shows that systemic inflammation in CF may cause an increase in arterial stiffness and recurrent exacerbations may increase the risk of CVD.

## 1. Introduction

Cystic fibrosis (CF) is a chronic progressive lung disease with systemic inflammation. Although there is an increased risk of cardiovascular disease (CVD) in chronic inflammatory lung and systemic inflammatory diseases (chronic obstructive pulmonary disease, rheumatoid arthritis, systemic vasculitis, inflammatory bowel disease, etc.), there is no clear evidence of CVD incidence in CF patients [1–3]. In a study by Hull et al. [4], it was shown that the presence of systemic inflammation in CF patients may cause vascular changes (increased arterial stiffness) and CVDs may be seen more frequently with prolonged survival. Also, systemic inflammation in patients is thought to cause impaired endothelial function, leading to an increase in arterial stiffness.

Augmentation index (Aix) and pulse wave velocity (PWV) are frequently used methods to evaluate arterial stiffness in recent years. They provide an early assessment of changes in the structure and function of large arteries and predict cardiovascular events [5]. These noninvasive and reproducible measurements are used to evaluate cardiovascular events and risk factors in many diseases such as CF.

The increase in CVD risk in chronic obstructive pulmonary disease exacerbation is related to the severity of respiratory tract infection and an increase in systemic inflammation [6]. In the study on vascular changes in acute exacerbation in adult CF patients, Aix was compared in exacerbation and after antibiotic treatment, and a significant reduction in Aix was demonstrated with treatment​ [7].

Our previous study demonstrated increased arterial stiffness in children with CF compared to healthy controls of similar age and sex. Although patients did not have common traditional risk factors (increased cholesterol and low-density lipoprotein, hypertension, and obesity), increased arterial stiffness was identified [8]. Acute exacerbations and chronic inflammation are risk factors for CVD in CF. This study aimed to investigate atherosclerosis and CVD in CF patients with acute pulmonary exacerbation. Of the markers of atherosclerosis, Aix, and pulse wave velocity (PWV) are associated with increased risk of CVD in acute exacerbation with increased systemic inflammation in CF. We hypothesized that there would be a decrease in hemodynamic measurements (risk of CVD) with a decrease in systemic inflammation with treatment and an increase in hemodynamic measurements with preincrement of the inflammation after treatment.

## 2. Materials and methods

### 2.1. Study design

The present study is an observational study using the general management in the Division of Pulmonology. All subjects provided written informed consent and the study was approved by the Local Research Ethics Committee (19-1T/41).

The study included patients between 3 and 18 years old who were followed up with the diagnosis of CF in the Ege University Pediatric Pulmonology Department and diagnosed with acute pulmonary exacerbation between January 15, 2019 and June 30, 2019. 

Patients who received antibiotic therapy for acute respiratory infection within 1 month, had pulmonary hypertension, required oxygen and continuous oxygen supply during infection, noninvasive mechanical ventilation, and systemic steroid treatment were excluded. CF-related diabetes was an exclusion factor because of one of the most important risk factors for the development of the cardiovascular disease.

Hemodynamic measurements were performed before and after treatment and 1 month after the end of treatment (clinically stable period) in patients who were planned for exacerbation treatment. C-reactive protein (CRP), fasting blood sugar, and pulmonary function tests (PFT) were evaluated from medical records and their relationship with hemodynamic measurements were evaluated. 

Bodyweight (kg) and height (cm) were measured. Body mass index (BMI) was calculated by weight (kg)/height (m2) formula. Measurements were reported as z-scores [9]. Shwacman–Kulczycki Score (SKS) was used to evaluate the clinical status of the patients [10]. The SKS calculated at the last visit in which patients were clinically stable was recorded from the medical records.

In our unit, the diagnosis of acute exacerbation in CF is based on anamnesis (increased cough, new or increased productive cough, change in sputum, hemoptysis, dyspnea, chest pain, and anorexia), physical examination (fever (body temperature > 38 °C), tachypnea, tachycardia, hypo-/hypertension, weight loss), laboratory findings (white blood cell, CRP), pulmonary function tests (>10% decrease in FEV1 and FVC). Intravenous antibiotic therapy decision is based on the antibiotic susceptibility of the microorganism produced in the previous sputum, induced sputum, throat, or bronchoalveolar lavage culture. According to the experience of the clinician, appropriate antibiotic therapy is initiated in patients without previous microorganism growth. Antibiotic treatment is often applied for 10–14 days for the causative or suspected microorganism.

Pulmonary function tests were performed by spirometry (Flowhandy ZAN 100, Germany) in accordance with American Thoracic Society standards. Forced vital capacity (FVC), forced expiratory volume in 1 s (FEV1) and flow rate in the middle of forced expiration (FEF25-75) were calculated as percent predicted values. The results of the patients who underwent PFTs for the diagnosis of acute exacerbation and the evaluation of treatment response were examined.

### 2.2. Hemodynamic measurements

Measurements were performed 2 h after food intake and before inhaler therapy (short-acting beta-2 agonist, dornase alpha, and inhaled antibiotics). All hemodynamic measurements were made under similar conditions (food intake, inhaler use, time of administration). Because of technical problems that may occur in hemodynamic measurements in individuals under 3 years old, patients over 3 years old were included.

Arterial stiffness measurements were performed in a quiet room while the patient had been resting for 15 min, in a sitting position. Mobil-O-Graph 24 h PWA Monitor (IEM GmbH, Stolberg, Germany) was used for the measurement. An appropriate cuff was chosen for the measurement of the brachial artery with the oscillometric method. A Bluetooth connection was established between the device and the software program developed for data analysis (Hypertension Management System; Client-Server Company, Stolberg, Germany; IEM GmbH). Age, sex, height, weight, and smoking information were entered into the software program. PWV analysis was performed three times at 5-min intervals. The data were classified as high-quality and low-quality by the software program. Only high-quality data were evaluated. The calculated pulse wave analysis was defined as aortic pulse wave. Aix heart rate was normalized to 75 beats/min to avoid interindividual variability secondary to heart rate. Measurements were performed by staff specifically trained in the technique and blinded to the clinical characteristics of each subject.

### 2.3. Statistical analysis

For statistical evaluation, the data were analyzed with SPSS version 20.0 software. Descriptive data were expressed as mean ± SD if analyzed with parametric tests and median (min–max); if analyzed with nonparametric tests, categorical variables were expressed by frequency and percentage. The Shapiro–Wilk test was used for the evaluation of normal distribution. A paired t-test was applied in parametric condition and the Wilcoxon test was used in nonparametric conditions in comparison of 2 repeated measurement data before and after treatment. Three repeated measurement data collected before treatment, after treatment, and during a stable period were compared with repeated measure ANOVA test. The correlation between Aix and PFTs and CRP were carried out by using the Pearson correlation if parametric test criteria were satisfied and by using the Spearman correlation analysis if not. In all comparisons, P < 0.05 was accepted as the limit of statistical significance. 

## 3. Results

Antibiotic treatment was initiated in 32 patients due to acute exacerbation. Four patients who needed oxygen and one patient from whom measurement could not be taken were excluded. Twenty-seven patients with hemodynamic measurements completed were included in the study. Demographic data of the patients are presented in Table 1.

**Table 1:** Clinical characteristics of the patients with CF.

	n = 27
Age (months) (median (min–max))	100 (36–211)
Sex (F/M)	16 (59.3) / 11 (40.7)
Z-score of weight (mean ± SD)	–1.58 ± 1.70
Z-score of height (mean ± SD)	–0.19 ± 1.97
Z-score of BMI (mean ± SD)	–2.34 ± 2.56
Mutation, n (%)	
F508del homozygous	10 (37)
F508del heterozygous	8 (29.6)
Others	9 (33.3)
Colonization, n (%)	
+/-	23 (85.2) / 4 (14.8)
Microorganism, n (%)	
Pseudomonas aeruginosa	23 (88.4)
Staphylococcus aureus	13 (73.9)
Aspergillus spp	1 (4)
Escherichia coli	3 (13)
Shwachman–Kulczycki scores (median (min–max))	60 (40–85)

BMI; body mass index, SD; standard deviation

Of the patients, 40.7% (n = 11) had inhaled short-acting beta-2 agonists and inhaled steroids, and 73.9% (n = 17) had inhaled antibiotic therapy. There were no patients on macrolide antibiotics. All patients were using dornase alpha inhaled therapy.

### 3.1. Acute exacerbation

The median duration of treatment was 14 (10–21) days. Microorganisms causing acute exacerbation were *Staphylococcus aureus* in 15 (55.5%) patients, *Pseudomonas aeruginosa* in 22 (81.4%) patients, *Escherichia coli* in 4 (14.8%) patients, *Aspergillus spp* in 2 (7.4%) and *Haemophilus influenza nontype* b in 2 (% 7.4) patients. Beta-lactam/lactamase group antibiotics were administered to 4 (14.8%) patients, cephalosporin to 6 (22.2%), carbapenem to 8 (29.6%), aminoglycoside to 20 (74%), glycopeptide to 11 (40.7%), fluoroquinolone to 9 (33.3%) patients, and antifungal therapy was initiated in 4 (14.8%) patients. 

There was no patient with fever during the measurements. No significant difference between peripheral oxygen saturation (SpO2) ​​before and after treatment was found (P > 0.05). While there was no statistically significant difference between systolic and diastolic blood pressures, a significant decrease in heart rate after treatment was noticed (P < 0.05). The CRP reduced after treatment (P < 0.01). Fasting blood glucose was similar before and after treatment (P > 0.05). Spirometry parameters (FEV1, FVC, and FEF25-75% predicted) showed a statistically significant increase after treatment (P < 0.05) (Table 2).

**Table 2:** Subject characteristics, pulmonary function tests, and laboratory measurements before and after treatment N: 27.

	Before treatmentmean ± SD	After treatmentmean ± SD	P**
Heart rate (bpm)	109.88 ± 24.24	99.29 ± 18.77	<0.050
Systolic BP (mm/Hg)	111.22 ± 12.50	108.96 ± 12.40	0.180
Diastolic BP (mm/Hg)	64.11 ± 11.52	63.29 ± 12.03	0.710
CRP (mg/dL)	1.80 (0.03-15.44)*	0.30 (0.03-3.77)*	<0.001***
Glucose (mg/dL)	92.00 ± 14.69	88.14 ± 13.57	0.230
Spirometry N:18			
FEV1, %	59.38 ± 24.01	73.27 ± 28.26	<0.001
FVC, %	56.22 ± 20.09	67.36 ± 24.19	<0.001
FEF25-75, %	65.50 ± 43.62	81.38 ± 44.80	0.020

BP; blood pressure, CRP; c reactive protein, FEV1; forced expiratory volume in 1 second, FVC; forced vital capacity, FEF25-75; forced expiratory flow during the middle half of FVC, SD; standard deviation, *median (min–max) for nonparametric distribution, **paired t-test, ***Wilcoxon test

### 3.2. Hemodynamic measurements

Hemodynamic measurements before and after treatment were evaluated in 27 patients. There was a statistically significant difference in Aix before and after treatment (44.25 ± 13.02% versus 33.48 ± 12.65%) (P < 0.01), but no significant difference in PWV (4.43 ± 0.37 m/s versus 4.40 ± 0.41 m/s) (P = 0.64).

Measurements were repeated in 21 patients who were clinically stable 1 month following acute exacerbation. While no statistically significant difference was found between PWV (P > 0.05), a statistically significant difference for Aix before and after treatment (P < 0.05), and before treatment and stable clinical condition (P < 0.05) was obtained (Figure). No statistically significant difference in heart rate and other hemodynamic measurements was found (P > 0.05) (Table 3). 

**Figure:**
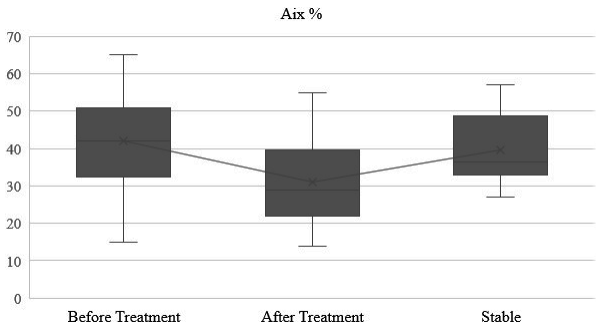
Augmentation index (Aix, %) before treatment, after treatment, and in clinically stable period.

**Table 3:** Hemodynamic measurements before treatment, after treatment, and in clinically stable period N: 21.

	Before treatmentmean ± SD	After treatmentmean ± SD	Stablemean ± SD	P*
Heart rate (bpm)	107.81 ± 5.31	98.09 ± 4.35	99.14 ± 5.12	0.790
Peripheral				
SBP (mm/Hg)	112.66 ± 12.65	109.14 ± 13.23	110.33 ± 13.53	0.310
DBP (mm/Hg)	64.14 ± 9.54	62.90 ± 11.51	67.14 ± 8.74	0.180
PP (mm/Hg)	48.52 ± 11.03	46.23 ± 11.36	43.19 ± 10.86	0.200
MAP (mm/Hg)	86.42 ± 9.56	83.95 ± 10.88	86.66 ± 9.84	0.250
Central				
SBP (mm/Hg)	97.57 ± 11.43	94.90 ± 11.89	99.04 ± 12.83	0.200
DBP (mm/Hg)	66.09 ± 9.44	64.90 ± 11.43	68.85 ± 8.84	0.210
PP (mm/Hg)	31.47 ± 7.80	30.00 ± 8.37	30.19 ± 9.42	0.790
AP (mm/Hg)	7.00 ± 3.60	5.76 ± 3.74	7.14 ± 5.12	0.440
Aix (%)	41.95 ± 12.96	30.95 ± 11.47	34.19 ± 14.36	0.010
PWV (m/s)	4.48 ± 0.39	4.39 ± 0.44	4.50 ± 0.47	0.330

Aix; augmentation index, AP; augmented pressure, DBP; diastolic blood pressure, MAP; mean arterial pressure, PP; pulse pressure, PWV; pulse wave velocity, SBP; systolic blood pressure SD; standard deviation. *repeated measure ANOVA

A significant positive correlation between pretreatment Aix and CRP (r = 0.40, P = 0.03), and a negative correlation between pretreatment Aix and FEV1% predicted (r = –0.74, P < 0.001), FVC% predicted (r = –0.73, P = 0.001) and FEF25-75% predicted (r = –0.56, P = 0.01) was found. There was no statistically significant correlation between age and hemodynamic measurements and changes in hemodynamic measurements before and after treatment (P > 0.05). Pretreatment Aix was correlated with a poor clinical condition (SKS) (r = –0.56, P = 0.002) and BMI z-score (r = –0.51, P = 0.006) before exacerbation. The change in Aix before and after treatment was not correlated with changes in CRP and PFTs (P > 0.05). The change in Aix was correlated with pretreatment Aix (r = –0.51, P = 0.006), FEV1% predicted (r = 0.55, P = 0.01), FVC% predicted (r = 0.55, P = 0.01) and SKS (r = 0.40, P = 0.03). 

## 4. Discussion

The decrease of arterial stiffness (Aix) with acute exacerbation treatment in children with CF has been demonstrated in the present study. This result shows that systemic inflammation in CF may cause an increase in arterial stiffness and recurrent pulmonary exacerbations may increase the risk of CVD. Furthermore, this is the first study to demonstrate increased arterial stiffness and associated risk of CVD in children with CF during acute exacerbations. Increased arterial stiffness is associated with poor clinical condition (PFTs, BMI, and clinical score) and systemic inflammation (CRP). 

Recent studies have shown an increased risk of CVD in chronic diseases associated with systemic inflammation [2,11]. Even in acute infections with increased inflammation, transient increases in the risk of CVD have been reported [12]. The mechanism by which systemic inflammation causes vascular changes is not fully known. Both localized airway inflammation and chronic systemic inflammation occur in CF. Increased circulating cytokines and chemokines, acute phase reactants are responsible for systemic inflammation [13]. Continuous polymorphonuclear cell (PMN) activation by recurrent pulmonary infections causes the release of granular myeloperoxidase (MPO). MPO has been shown to cause an increase in circulating proinflammatory cytokines [14,15]. In addition, PMNs activated during exacerbations give rise to an increase in reactive oxygen species (ROS). All these systemic inflammatory effects and increased oxidative stress are thought to cause changes in vascular functions [14]. Increased markers of systemic inflammation and ROS products in acute exacerbations have been shown to decrease with treatment [16]. Although there is a decrease in inflammation products with intensive treatment, there is no complete improvement due to chronic inflammation [17]. In this study, in agreement with this information, Aix has been shown to decrease with systemic inflammation after acute exacerbation treatment. In a study of adult population, evaluating the effects of acute exacerbation treatment on hemodynamic measurements and CVD risk in CF patients, a statistically significant decrease in Aix (10.9 ± 10.9% versus 8.1 ± 10.9%) with improvement in clinical status and PFTs was shown with treatment. However, the change in hemodynamic measurements after the termination of the efficacy of treatment has not been studied [7]. To the best of our knowledge, there are no other studies of similar design. In this study, it is thought that there could be an increase in Aix due to chronic inflammation continuing after treatment. Although the hemodynamic measurements of the patients in the stable clinical period were increased, that change was not statistically significant. That insignificance might have stemmed from remeasurement shortly after infection. Hence, a statistically significant increase might also be observed with measurements taken after a longer period. 

Increased arterial stiffness is an independent risk factor for CVD. Studies have been conducted and used to evaluate CVD risk in several diseases. Arterial stiffness can be determined by aortic PWV, the direct measurement method, or by Aix, the measurement of the composition of complex and wave reflections [18,19]. Arterial stiffness and wave reflections are affected differently by age and various clinical conditions. In this study, there was no statistically significant correlation between age and hemodynamic measurements. The different clinical conditions and severity of infection of the patients may cause this condition. Age-related changes were more prominent in PWV for individuals over the age of 50, and in Aix for individuals under the age of 50 [20]. In a study conducted in patients with rheumatoid arthritis over 50 years, PWV was found to be significantly higher than in healthy controls, whereas, in another study performed in patients under 50 years, PWV was similar while Aix were significantly higher than healthy controls [21,22]. Consistent with this information, in our study of pediatric patients, no statistically significant change was found in PWV after treatment and a significant change was obtained in Aix. 

Treatment strategies for systemic inflammatory diseases are planned to reduce inflammation. The risk of CVD has been observed to reduce with the decrease of inflammation [23]. Even in healthy individuals, acute phase reactants such as CRP are predictive markers for CVD [24]. In a study of patients with systemic vasculitis, PWV, Aix, and CRP were found to be significantly higher in the active disease group compared to healthy controls. Hemodynamic measurements showed a positive correlation with CPR. While Aix was significantly higher in active disease than in remission patients, PWV was shown to be similar [25]. In a study, hemodynamic measurements of CF patients were compared with those of healthy controls, and Aix was found to be higher in CF patients and it was found to be positively correlated with CRP. In another study examining the change in hemodynamic measurements with acute infection treatment, no correlation was found between the change in Aix and change in CRP after treatment [4,7]. In the present study, there was a significant positive correlation between pretreatment Aix and CRP (r = 0.40, P = 0.03). There was no statistically significant correlation between the change in Aix and CRP before and after treatment (P > 0.05).

The decrease in arterial stiffness after treatment of acute exacerbation can be explained by improvement in clinical status with a decrease in systemic inflammation and improvement in PFTs. In a study examining the effects of atorvastatin on arterial stiffness in patients with rheumatoid arthritis, a significant reduction in arterial stiffness was demonstrated after 6 weeks of treatment. The greatest change in Aix has been reported in patients with high scores of disease activity [26]. In another study investigating the effect of antitumor necrosis factor-alpha on arterial stiffness, a significant decrease in aortic PWV was observed together with the decrease in disease activity score and inflammation markers (CRP and erythrocyte sedimentation rate) after 12 weeks of treatment [21]. Pulmonary function tests play an important role in determining the severity of acute exacerbation and lung injury in CF. FEV1 provides information on the severity and clinical status of the disease. The Shwachman–Kulczycki score is a scoring system used to monitor the severity of the disease, especially in patients with pulmonary dysfunction. There was a significant negative correlation between pretreatment Aix and PFTs and a positive correlation between pretreatment Aix and CRP. Pretreatment Aix was associated with a poor clinical condition and BMI before exacerbation. The mean increase in FEV1% predicted after antibiotic treatment was 14.22%. The change in Aix was associated with pretreatment Aix (r = –0.51, P = 0.006), FEV1% predicted (r = 0.55, P = 0.01), FVC% predicted (r = 0.55, P = 0.01) and SKS (r = 0.40, P = 0.03). Although the mechanisms of action that may cause a decrease in Aix have not been studied in the study, a decrease in inflammation, an increase in PFTs, and improvement in clinical conditions are thought to be potentially effective. 

### 4.1. Limitations

This study aimed to investigate the changes in hemodynamic measurements in acute exacerbation in children with CF. The mechanisms of action that could lead to changes in hemodynamic measurements were not evaluated.

Antibiotic decision and treatment duration were decided based on the microorganism that cause or is thought to cause infection, and the clinician’s experience. It should be kept in mind that the choice of empirical treatment in patients with unknown etiology may affect the change in hemodynamic measurements. In addition, the effect of antibiotic changes and treatment duration made by the clinician according to culture results and clinical response on hemodynamic measurements was not examined. The standard treatment regimens for future studies should be determined considering all these differences.

Pulmonary function tests were performed in small number of patients due to patient-related factors, technical problems and the high proportion of patients at a young age in 1 month after the treatment of acute exacerbation. Therefore, the relationship between hemodynamic measurements and PFTs were not discussed.

In conclusion, this is the first study to investigate the effects of acute exacerbation treatment on vascular changes and CVD risk in children with CF. Here hemodynamic measurements have been shown to decrease with antibiotic treatment. Prevention and treatment of acute exacerbations in childhood may reduce the risk of CVD in patients. Large-scale, prospective studies are required to evaluate the effects of vascular changes emerging in childhood on CVD in further ages. In addition, our study shows that hemodynamic measurements are related to parameters (SKS, PFTs, and BMI, etc.) used in the evaluation and follow-up of patients with CF. This study may be a pioneering study for the use of hemodynamic measurements in the routine follow-up of these patients.

## Acknowledgments

The authors thank the patients, and laboratory staff member Gökay Çelik for spirometry and Filiz Kaygısızer Keleş for technical assistance. Additionally, we would like to express our special thanks to Prof. Dr. Pembe Keskinoğlu from Dokuz Eylül University Faculty of Medicine Department of Basic Medical Sciences, Department of Biostatistics and Medical Informatics for valuable contributions to the statistical analysis of the study.

## Conflict of interest

The authors declare that there is no conflict of interest.
